# Minimally invasive laminar lift and posterior cervical laminoplasty via the intermuscular approach: a canine model study

**DOI:** 10.1590/acb370903

**Published:** 2022-11-28

**Authors:** Fei Yan, Zejian Jin, Yuhong Song, Yinghao Liu, Yonggang Wang, Lei Miao, Bin Liu, Hetian Song

**Affiliations:** 1 MSc. Tongliao City Hospital – Fourth Department of Orthopedic – Tongliao (Inner Mongolia), China.; 2 BSc. Tongliao City Hospital – Department of Radiology – Tongliao (Inner Mongolia), China.; 3 BSc. Tongliao Mengdong Mongolian Medical Hospital – Tongliao (Inner Mongolia), China.; 4 BSc. Tongliao City Hospital – Fourth Department of Orthopedic – Tongliao (Inner Mongolia), China.

**Keywords:** Laminoplasty, Minimally Invasive Surgical Procedures, Dogs

## Abstract

**Purpose:**

This study aimed to develop a minimally invasive surgical procedure for laminar lift and posterior cervical laminoplasty via the intermuscular approach using a canine model.

**Methods:**

Six Alaskan dogs were used for developing the surgical approach. The bilateral laminae of C3-7 were cut with an ultrasonic osteotome and fixed with bilateral plates to maintain the lamina lifting and reshape a wider spinal canal. The important structures, such as ligaments, supraspinous ligaments, interspinous ligaments, and ligamentum flavum were preserved. The therapeutic effect was evaluated by preoperative and postoperative imaging results and neck mobility.

**Results:**

The surgical procedures were all successfully performed in the 6 animals. All the dogs survived well within 1 year of postoperative follow-up. The postoperative neck mobility was as good as the preoperative one. Computed tomography results showed that the anteroposterior diameter of the spinal canal was successfully enlarged and maintained well.

**Conclusions:**

The minimally invasive surgical procedure for laminar lift and posterior cervical laminoplasty via the intermuscular approach was feasible in a canine model, which might be applied in clinical practice.

## Introduction

Cervical spondylotic myelopathy (CSM) is a degenerative disease causing progressive stenosis of the cervical spinal canal and subsequent compression of the spinal cord and/or surrounding blood vessels^1^^,^ 2. The compression can trigger a series of pathophysiological processes, such as ischemia, inflammation, neuronal apoptosis at the site of compression, producing cervical myelopathy^3^. CSM is the most common form of nontraumatic spinal cord injury in adults aged over 55 years^4^^,^ 5, accounting for 10% to 15% of all types of cervical spondylosis^6^. It may cause long-term disability and major neurological impairments, severely affecting patients’ quality of life^7^.

At present, the mainstream management of multilevel CSM (MCSM) aims to identify early symptoms and provide effective treatment before development of irreversible spinal cord damage^8^. The MCSM patients should receive surgical treatment within 3–6 months after the onset of the disease to decompress the spinal cord and nerves, which can prevent the progression of spinal cord injury and provide favorable conditions for the recovery of nerve function^9^. Posterior cervical laminoplasty was developed by Hirabayashi *et al.* 10, which can effectively enlarge the anterior and posterior diameter of the spinal canal, thereby relieving cord compression, improving the blood supply, and reducing the interference to the cervical spine mobility^11^. Posterior cervical laminoplasty overcomes the shortcomings of cervical kyphosis following cervical laminectomy, and can reduce operation time, hospital stay, and surgical risk, preserving good cervical spine mobility and postoperative function^12^^–^ 16. This surgical procedure has been applied to the treatment of MCSM and achieved good therapeutic outcomes^17^^–^ 20. Posterior cervical laminoplasty has been widely used to treat MCSM with confirmed therapeutic efficacy.

However, posterior cervical laminoplasty has been reported to induce a high incidence of postoperative axial symptoms (such as pain and stiffness)^21^^–^ 25, which reduce significantly patient satisfaction. The etiological mechanisms of postoperative axial symptoms following posterior cervical laminoplasty have not been fully understood, but are thought to be related to surgical injury to the posterior cervical spinous process-ligament-muscle complex^26^^,^ 27. Therefore, it is urgently required to develop a surgical method to reduce postoperative complications of posterior cervical surgery.

The purpose of this study was to develop a minimally invasive surgical procedure for laminar lift and posterior cervical laminoplasty via the intermuscular approach using a canine model, which can fully protect the posterior cervical spinous process-ligament-muscle complex.

## Methods

### Animals

Six healthy Alaskan dogs (aged 2–3 years, weighing approximately 45 kg) with strong physique and thick bones were purchased from local pet institutions. The 6 dogs were examined, injected with relevant vaccines, dewormed, and cleaned. The 6 dogs were raised in the same suitable environment. All protocols used in this study were approved by the Institutional Animal Care and Use Committees (IACUCs) of the Tongliao City hospital.

### Preoperative imaging examinations

The dog was injected with atropine (0.1 mg/kg) and tiletamine hydrochloride and zolazepam hydrochloride (Zoletil, 10 mg/kg). After the drug took effect, the animal was put onto the examination instruments for preoperative imaging examinations, including digital radiography (DR), computed tomography (CT), and magnetic resonance imaging (MRI). After the examination, the experimental dog was injected with anesthesia antagonist (Suxingling, 0.1 mL/kg) and was returned to the breeding place after fully awake.

### Surgical procedure

In the preoperative preparation, the day before the operation, the animal was washed and weighed, and the surgical area of the neck was shaved. The animal was fasted for 12 h and water for 4 h before surgery. Before entering the operating room, the dog was injected intramuscularly with tiletamine hydrochloride and zolazepam hydrochloride (10 mg/kg), and an intravenous indwelling needle was placed. The dog was injected with atropine (0.04 mg/kg) intravenously, and antibiotics and sedatives were infused by intravenous drip. After 30 min, the dog was placed on a sterile animal operating bed, and received induction anesthesia (tiletamine hydrochloride and zolazepam hydrochloride, 10 mg/kg; Zoletil, Virbac, USA) and general anesthesia. The maintenance anesthesia drug was sevoflurane at a maintenance concentration of 2%. The dog received endotracheal intubation that was connected to an anesthesia machine, and the vital signs were closely monitored.

**Figure 1:**
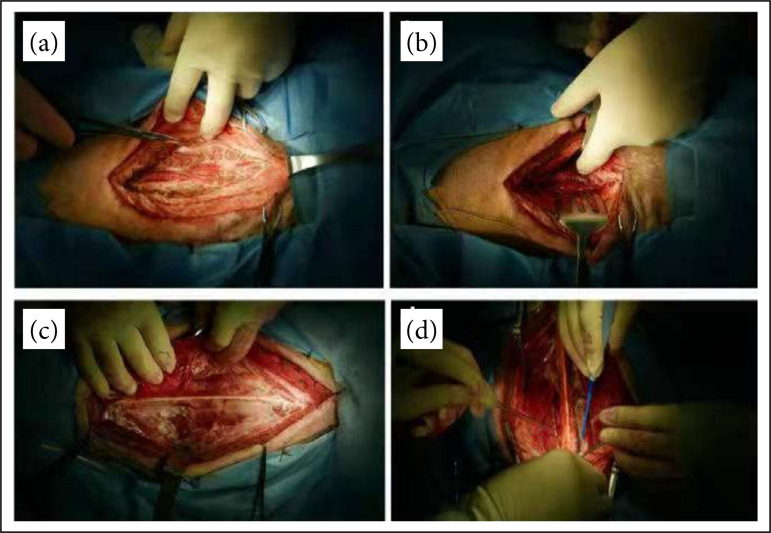
After subcutaneous tissue was separated, the posteriordermatome, platysma, clavicularis, trapezius, rhomboid,were sequentially incised **(a** and **b)** and separated toboth sides along the nuchal ligament **(c** and **d)**.

**Figure 2:**
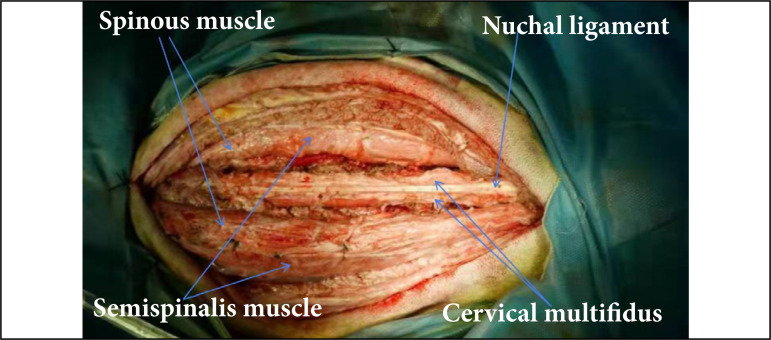
The operation was performed through the spacebetween the cervical multifidus and the spinous/semispinalismuscles (intermuscular approach).

**Figure 3:**
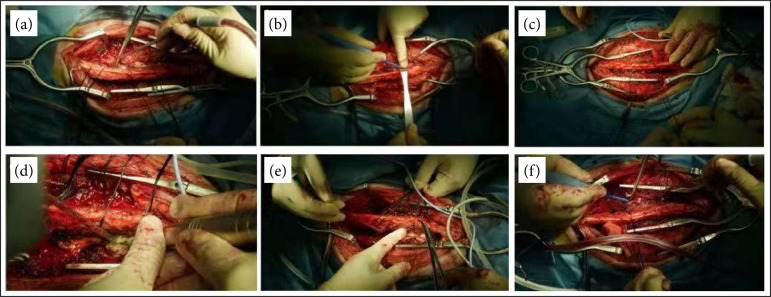
The cervical multifidus was pulled medially, and the spinous and semispinalis muscleswere pulled laterally, completely exposing bilateral cervical 3–7 laminae to the inner edge of thearticular process **(a–d)**. The supraspinous ligament, interspinous ligament, and ligamentumflavum were preserved. The bilateral laminae were cut by an ultrasound **(e and f)**.

**Figure 4:**
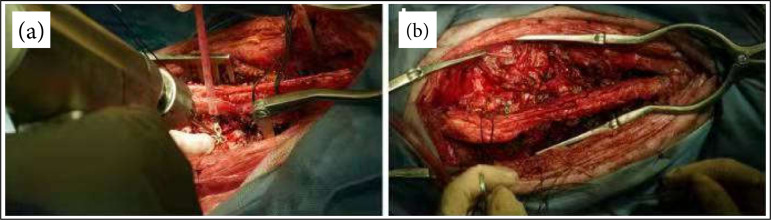
The severed lamina was stretched by 5 mm with **(a)** a hand surgical peeler, and**(b)** hand surgical steel plates were placed on the bilateral C3-C7 laminae.

The dog was placed in the prone position, and the head and waist were fixed with restraint belts, and the surgical area (from the back of both ears to the bilateral costal margins) was sterilized with iodophor. A longitudinal incision (about 25 cm) was made from the occipital tubercle to the T1 spinous process. After subcutaneous tissue was separated, the posterior dermatome, platysma, clavicularis, trapezius, rhomboid, were sequentially incised (Fig. 1a,b) and separated to both sides along the nuchal ligament (Fig. 1c,d). The operation was performed through the space between the cervical multifidus and the spinous/semispinalis muscles (Fig. 2). The cervical multifidus was pulled medially, and the spinous and semispinalis muscles were pulled laterally, fully exposing the root of the spinous process of the 3–7 lamina of the bilateral cervical vertebrae to the inner edge of the articular process (Fig. 3a–d). The supraspinous ligament, interspinous ligament, and ligamentum flavum were preserved. The bilateral laminae were cut by an ultrasound osteotome (Model XD860A, Jiangsu Shuimu Tianpeng Medical Technology Co., China) along the medial borders of the bilateral articular processes (Fig. 3e,f). The severed lamina was stretched by 5 mm with a hand surgical peeler. A 2.0-mm T-shaped hand surgical steel plate (CITIC Industrial Fund Dingjian Medical Instrument Co., China) was trimmed and placed on the bilateral C3–C7 laminae (Fig. 4a,b). The plates should be placed mid-superior to the inferior articular process and the screws should be accurately measured to avoid the screw penetration of the zygapophyseal joint. Two 2.0-mm screws were fixed on the laminar side of the steel plate, and two 2.0-mm screws were fixed on the articular process side of the plate (the lamina of the bilateral cervical 3 are small, and this lamina side can only be fixed with 1 screw). The screws were used to fix the plate to maintain the elevation of the lamina and the enlargement of the spinal canal. After confirming that the internal fixation was firmed, the incision was sutured.

### Postoperative management

The dog was treated with cefazolin (1.0 g two times a day) for anti-infection and parecoxib (20 mg two times a day) for analgesia within 3 days after surgery. At 2 weeks after operation, the dogs were examined for neck mobility. After sedation, the incision was disinfected and sutures were removed. The dogs were then examined with DR, CT, and MRI. Neck mobility and imaging examinations were re-performed 3 months and 1 year after the operation.

According to the imaging data, the anterior and posterior diameters of the C3–C7 spinal canal were compared before and after the operation. The preoperative and postoperative neck mobility were also compared.

## Results

The surgical procedures were all successfully performed in the 6 animals. All the dogs survived well within 1 year of postoperative follow-up. The postoperative neck mobility was good; without significant change as compared with the preoperative one.

**Figure 5:**
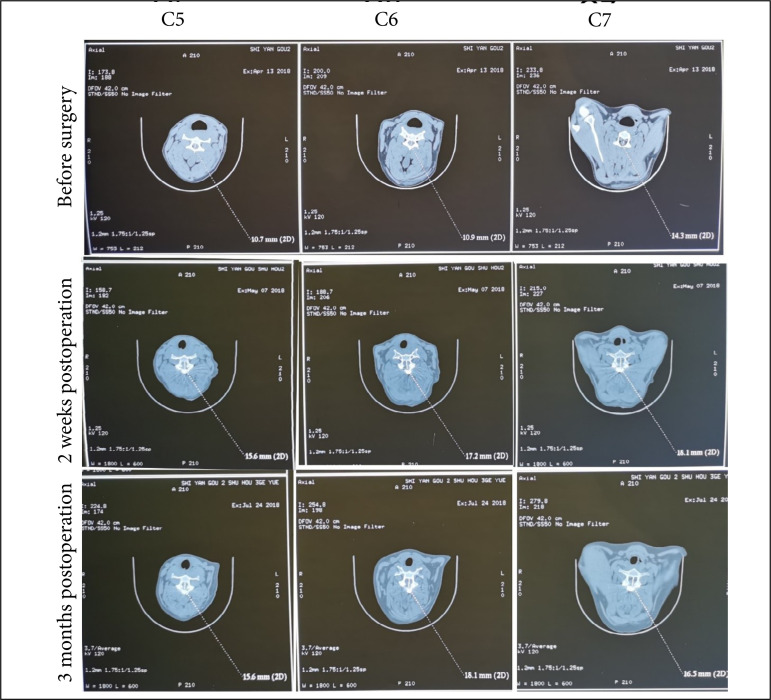
The CT measurement on the anteroposterior diameter of the spinalcanal (C5–C7) before surgery and at 2 weeks and 3 months after operation.

**Figure 6:**
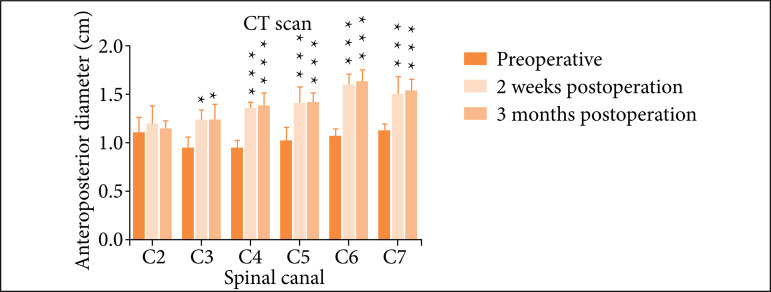
Comparison of CT measurement on the anteroposterior diameter of thespinal canal (C3–C7) before surgery and at 2 weeks and 3 months after operation.

There are many artifacts in postoperative MRI (data not shown). The clarity of CT images was better than MRI images, hence CT was used as the postoperative evaluation method. CT results showed that the anteroposterior diameter of the spinal canal (C3–C7) was significantly enlarged and maintained well at 2 weeks and 3 months after operation as compared with preoperative measurement (Figs. 5 and 6, all p < 0.05).

*p < 0.05; ***p < 0.001, compared with preoperative data.

## Discussion

The axial symptoms following posterior cervical laminoplasty are related to the posterior cervical muscle atrophy and the surgical injury to the ligament complex^28^. In the conventional cervical posterior single-door laminoplasty, to ensure inversion of the C3 lamina, all or most of the cervical semispinalis muscle on the C2 spinous process needs to be dissected, and the interspinous and supraspinous ligaments between C2 and C3, C6 and C7 laminae should be cut off. If the stripped cervical semispinalis muscle is not completely repaired at the end of the operation, the cervical semispinalis muscle will atrophy, the muscle and ligament system will be injured, which affects the stability of the cervical spine, leading to postoperative axial symptoms^29^. In addition, decreased cervical mobility due to surgical scars-induced adhesion between muscles and ligaments of the posterior cervical muscles can also result in axial symptoms. In the conventional cervical posterior single-door laminoplasty, when dealing with the opening side and the hinge side of the lamina, the adjacent facet joints, joint capsules and articular surface could be damaged to varying degrees. Suspension of the lamina from the hinge-side facet capsule or paravertebral tissue may injure the sensory nerve endings of the posterior rami of the cervical nerve. Meanwhile, the suspension suture-induced traction injury of the facet joint capsule and the paravertebral soft tissue can lead to aseptic inflammation, and then form scar tissue, which limits the mobility of the facet joints. All of the above conditions can lead to muscle spasm and pain in the neck, shoulders, and back^30^. A retrospective study by Chen *et al.* 31 also showed that the incidence and severity of postoperative axial symptoms were closely related to the number and degree of destruction of the facet joints.

In the posterior cervical laminoplasty via the intermuscular approach described in this study, the posterior dermatome, platysma, clavicularis, trapezius, rhomboid, and splenius to the nuchal ligament were separated in turn, and the bilateral ligaments were separated along with the space between the cervical multifidus, the spinous muscle, and the semispinalis muscle. This surgical approach can completely preserve the important posterior cervical structures, such as the spinous process, supraspinous ligament, interspinous ligament, ligamentum flavum, nuchal ligament, and muscle attachment points, avoiding scar tissue formation. The posterior cervical spinous process-ligament-muscle complex was completely preserved. The C3–C7 laminae were fixed with bilateral steel plates, so that the enlargement of the spinal canal can be stably maintained even at 3 months after operation. The bilateral decompression of the spinal cord is more sufficient, and the surgery is minimally invasive. The overall backward movement of the spinous process-ligament-muscle complex was achieved within its elastic range, preventing excessive volume expansion and nerve root traction syndrome.

Ultrasonic osteotome is an efficient surgical device with advantages, such as high tissue selectivity, high osteotomy accuracy, low working temperature, good antirolling, minor damage to peripheral blood vessels and nerve tissue, and hemostasis while cutting^32^. In this study, the cervical laminae were cut with an ultrasonic osteotome with saline perfusion to minimize the damage to the spinal cord. During the operation, the ultrasonic osteotome should be avoided to stay in the same part for a long time to reduce the possibility of dura and nerve damage. When cutting the lamina, the right hand controlled the movement of the ultrasonic osteotome, and the left hand should maintain an upward-lifting force to prevent the right hand from exerting too much force. This can maintain the vibrating state of the ultrasonic osteotome, reducing the risk of thermal damage to surrounding tissues and the risk of damage to the spinal cord and dura. When the ultrasonic osteotome is close to the nerve and vascular plexus, it should be operated with caution to avoid accidental injury. Thus, the stimulation of the facet joint capsule in the traditional fixation method can be avoided, reducing the incidence of axial symptoms. In addition, the cervical spine was not fused, so that the motility of the cervical spine was not affected.

In summary, this study demonstrated that minimally invasive laminar lift and posterior cervical laminoplasty via the intermuscular approach are feasible in a canine model, and, the plates were firmly fixed, and the surgical effect is good. This could provide a safe and feasible surgical method for the minimally invasive treatment of MCSM. Future studies should be conducted to perform this procedure in cadaver experiments, and design more appropriate internal fixation materials.

## References

[1] Oliveira Vilaça C, Orsini M, Leite MAA, Freitas MRG, Davidovich E, Fiorelli R, Fiorelli S, Fiorelli C, Oliveira AB, Pessoa BL (2016). Cervical spondylotic myelopathy: What the neurologist should know. Neurol Int.

[2] Aljuboori Z, Boakye M. (2019). The natural history of cervical spondylotic myelopathy and ossification of the posterior longitudinal ligament: A review article. Cureus.

[3] Badhiwala JH, Ahuja CS, Akbar MA, Witiw CD, Nassiri F, Furlan JC, Curt A, Wilson JR, Fehlings MG. (2020). Degenerative cervical myelopathy — Update and future directions. Nat Rev Neurol.

[4] Iyer A, Azad TD, Tharin S. (2016). Cervical spondylotic myelopathy. Clin Spine Surg.

[5] Bakhsheshian J, Mehta VA, Liu JC (2017). Current diagnosis and management of cervical spondylotic myelopathy. Global Spine J..

[6] Feng S, Zheng B, Zhang L, Wang W. (2021). A systematic review and meta-analysis compare surgical treatment and conservative treatment in patients with cervical spondylotic myelopathy. Ann Palliat Med.

[7] Theodore N (2020). Degenerative Cervical Spondylosis. N Engl J Med..

[8] McCormick JR, Sama AJ, Schiller NC, Butler AJ, Donnally CJ. (2020). Cervical spondylotic myelopathy: A guide to diagnosis and management. J Am Board Fam Med.

[9] Melancia JL, Francisco AF, Antunes JL (2014). Spinal stenosis. Handb Clin Neurol.

[10] Hirabayashi K, Watanabe K, Wakano K, Suzuki N, Satomi K, Ishii Y. (1983). Expansive open-door laminoplasty for cervical spinal stenotic myelopathy. Spine (Phila Pa 1976).

[11] Cuéllar JM, Bae H. (2017). Posterior cervical laminoplasty. Oper Tech Spine Surg.

[12] Yeh KT, Chen IH, Yu TC, Liu KL, Peng CH, Wang JH, Lee RP, Wu WT (2015). Modified expansive open-door laminoplasty technique improved postoperative neck pain and cervical range of motion. J Formos Med Assoc.

[13] Hu W, Shen X, Sun T, Zhang X, Cui Z, Wan J. (2014). Laminar reclosure after single open-door laminoplasty using titanium miniplates versus suture anchors. Orthopedics.

[14] Wang M, Luo XJ, Deng QX, Li JH, Wang N. (2016). Prevalence of axial symptoms after posterior cervical decompression: A meta-analysis. Eur Spine J..

[15] Motosuneya T, Maruyama T, Yamada H, Tsuzuki N, Sakai H. (2011). Long-term results of tension-band laminoplasty for cervical stenotic myelopathy: A ten-year follow-up. J Bone Joint Surg Br.

[16] Duetzmann S, Cole T, Ratliff JK (2015). Cervical laminoplasty developments and trends, 2003-2013: A systematic review. J Neurosurg Spine.

[17] Yang SC, Niu CC, Chen WJ, Wu CH, Yu SW. (2008). Open-door laminoplasty for multilevel cervical spondylotic myelopathy: Good outcome in 12 patients using suture anchor fixation. Acta Orthop.

[18] Bhatia NN, Lopez G, Geck M, Gottlieb J, Eismont F (2015). Posterior cervical laminoplasty in the North American Population: A minimum of two year follow-up. Clin Neurol Neurosurg.

[19] Hardman J, Graf O, Kouloumberis PE, Gao WH, Chan M, Roitberg BZ (2010). Clinical and functional outcomes of laminoplasty and laminectomy. Neurol Res..

[20] Chen H, Liu H, Meng Y, Wang B, Gong Q, Song Y. (2018). Short-term outcomes of anterior fusion–nonfusion hybrid surgery versus posterior cervical laminoplasty in the treatment of multilevel cervical spondylotic myelopathy. World Neurosurg.

[21] Hale JJ, Gruson KI, Spivak JM. (2006). Laminoplasty: A review of its role in compressive cervical myelopathy. Spine J.

[22] Wang SJ, Jiang SD, Jiang LS, Dai LY (2011). Axial pain after posterior cervical spine surgery: A systematic review.. Eur Spine J..

[23] Chen C, Li J, Liao Z, Gao Y, Shao Z, Yang C. (2020). C3 laminectomy combined with modified unilateral laminoplasty and in situ reconstruction of the midline structures maintained cervical sagittal balance: A retrospective matched-pair case-control study. Spine J..

[24] Kong QJ, Luo X, Tan Y, Sun JC, Wang Y, Tan L, Shi JG. (2021). Anterior controllable antedisplacement and fusion (ACAF) vs posterior laminoplasty for multilevel severe cervical ossification of the posterior longitudinal ligament: Retrospective study based on a two-year follow-up. Orthop Surg.

[25] Yang HY, Zhang YG, Zhao D, Sun GM, Ma Y, Hao YH, Yang Q. (2021). A new posterior extensor attachment-point reconstruction technique for cervical spondylotic myelopathy involving C2 segment: Clinical outcome and safety. J Neurol Surg A Cent Eur Neurosurg.

[26] Okada M, Minamide A, Endo T, Yoshida M, Kawakami M, Ando M, Hashizume H, Nakagawa Y, Maio K. (2009). A prospective randomized study of clinical outcomes in patients with cervical compressive myelopathy treated with open-door or French-door laminoplasty. Spine (Phila Pa 1976).

[27] Sun Y, Zhang F, Wang S, Zhang L, Pan S, Yu M, Qiu S. (2010). Open door expansive laminoplasty and postoperative axial symptoms: A comparative study between two different procedures. Evid Based Spine Care J..

[28] Kimura A, Endo T, Inoue H, Seichi A, Takeshita K. (2015). Impact of axial neck pain on quality of life after laminoplasty. Spine (Phila Pa 1976).

[29] Riew K, Raich A, Dettori J, Heller J. (2013). Neck pain following cervical laminoplasty: Does preservation of the C2 muscle attachments and/or C7 matter?. Evid Based Spine Care J..

[30] Schneider GM, Jull G, Thomas K, Smith A, Emery C, Faris P, Schneider K, Salo P. (2013). Intrarater and interrater reliability of select clinical tests in patients referred for diagnostic facet joint blocks in the cervical spine. Arch Phys Med Rehabil..

[31] Chen H, Liu H, Li T, Gong Q, Song Y, Zheng J, Liu L, Kong Q. (2013). [Effect of penetration of mini-plate lateral mass screws into facet joint on axial symptoms in cervical laminoplasty]. Zhongguo Xiu Fu Chong Jian Wai Ke Za Zhi.

[32] Yu L, Wen JK, Wang S, Wang WH, Yu JM, Ye XJ. (2020). Removal of calcified lumbar disc herniation with endoscopic-matched ultrasonic osteotome–Our preliminary experience. Br J Neurosurg.

